# Chemical Composition and Antibacterial and Antioxidant Activity of a Citrus Essential Oil and Its Fractions

**DOI:** 10.3390/molecules26102888

**Published:** 2021-05-13

**Authors:** Carmen M. S. Ambrosio, Gloria L. Diaz-Arenas, Leidy P. A. Agudelo, Elena Stashenko, Carmen J. Contreras-Castillo, Eduardo M. da Gloria

**Affiliations:** 1 Dirección de Investigación y Desarrollo, Universidad Privada del Norte (UPN), 13001 Trujillo, Peru; 2 Research Center of Excellence CENIVAM, CIBIMOL, Industrial University of Santander, 680002 Bucaramanga, Colombia; gloriarussi92@gmail.com (G.L.D.-A.); elena@tucan.uis.edu.co (E.S.); 3 Khymos S.A, 111121 Bogota, Colombia; leidypaolaacevedo@gmail.com; 4 Department of Agri-Food Industry, Food and Nutrition, ESALQ, University of São Paulo, Piracicaba, 13418-900 São Paulo, Brazil; ccastill@usp.br; 5 Department of Biological Science, Luiz de Queiroz” College of Agriculture, University of São Paulo, Piracicaba, 13418-900 São Paulo, Brazil

**Keywords:** fractional distillation, carvone, *cis*/*trans*-carveol, limonene, *E. coli*

## Abstract

Essential oils (EOs) from *Citrus* are the main by-product of *Citrus*-processing industries. In addition to food/beverage and cosmetic applications, citrus EOs could also potentially be used as an alternative to antibiotics in food-producing animals. A commercial citrus EO—Brazilian Orange Terpenes (BOT)—was fractionated by vacuum fractional distillation to separate BOT into various fractions: F1, F2, F3, and F4. Next, the chemical composition and biological activities of BOT and its fractions were characterized. Results showed the three first fractions had a high relative amount of limonene (≥10.86), even higher than the whole BOT. Conversely, F4 presented a larger relative amount of BOT’s minor compounds (carvone, *cis*-carveol, *trans*-carveol, *cis*-p-Mentha-2,8-dien-1-ol, and *trans*-p-Mentha-2,8-dien-1-ol) and a very low relative amount of limonene (0.08–0.13). Antibacterial activity results showed F4 was the only fraction exhibiting this activity, which was selective and higher activity on a pathogenic bacterium (*E. coli*) than on a beneficial bacterium (*Lactobacillus* sp.). However, F4 activity was lower than BOT. Similarly, F4 displayed the highest antioxidant activity among fractions (equivalent to BOT). These results indicated that probably those minor compounds that detected in F4 would be more involved in conferring the biological activities for this fraction and consequently for the whole BOT, instead of the major compound, limonene, playing this role exclusively.

## 1. Introduction

*Citrus* are the most grow fruit around the world, and their processing results in a large amount of waste (peel, seeds, and pulp) and by-products [1]. Essential oils (EO) are the main by-product of *Citrus* processing industries, and they have been the most popular source of flavors (aromas) and fragrances of commercial value, principally for the cosmetic, food, and beverage industries [2,3]. This is due to citrus EOs being categorized and generally recognized as safe (GRAS) by the United State-Food and Drug Administration [4] and because they harbor several biological properties, which have turned them into an economic, eco-friendly, and natural alternative to synthetic preservatives and antioxidants for these industries [5].

A citrus EO is a mixture of more than 200 compounds, of which 85–99% are volatile compounds and 1–15% are non-volatile compounds [6]. The volatiles are mainly monoterpene and sesquiterpene hydrocarbons, their oxygenated derivatives, aliphatic aldehydes, alcohols, and esters. The non-volatile portion is comprised of hydrocarbons, sterols, fatty acids, waxes, carotenoids, coumarins, psoralens, and flavonoids [2]. However, the monoterpene limonene is the major compound in citrus EOs and may range from 32 to 98% [5]. The qualitative and quantitative composition of citrus EOs directly determines their features and biological properties [1], such as their antimicrobial and antioxidant activities. Some studies have indicated that possibly the major compound of citrus EOs, limonene, could be responsible for conferring the biological properties of these oils [7,8], but other authors have suggested that the consortia between major and minor components of EOs would be involved in conferring their biological properties [9].

In addition to the applications mentioned, citrus EOs could also have another potential application related to meeting the urgent demand for alternatives to substitute antibiotics in food-producing animals, such as pigs. During the last 15 years, there has been heightened concern about the impact on public health of the growing emergency of antibiotic-resistance superbugs, since the overuse of antibiotics in the raising of food-producing animals has been suspected as one of the main factors contributing to this matter [10]. As a measurement to counter this concern, antibiotics use, and more specifically, their dietary application as growth promoters in food-producing animals, has been banned within the European Union since 2006 [11]. Nonetheless, recently, some other countries have adopted restrictions regarding the use of some antibiotics for this purpose, such as China, Brazil, Japan, South Korea, New Zealand [12,13,14,15], and United States [16]. Interestingly, a feature that might distinguish citrus EOs as a promising alternative is their reported potential selective antibacterial activity, presenting a stronger antibacterial action on pathogenic than on beneficial bacteria that occur in the pig gut [17,18]. Selectivity toward pathogenic bacteria by EOs rather than beneficial bacteria has been highlighted as a desirable antimicrobial spectrum feature for candidate compounds as possible alternatives to antibiotics [19,20,21]. In addition, dietary supplementation with EOs could be a nutritional strategy to prevent oxidative stress and damage to the organism of food-producing animals, since it is well known that EOs harbor sources of natural antioxidants that can effectively inhibit oxidative reactions [22], thus preventing the formation of free radicals at localized sites of the animal organism and also on the derived animal products [19,22]. In this context, understanding what compounds contained in citrus EOs are associated with their selective antibacterial activity and antioxidant capacity is critical to their future development.

Fractional distillation is a unit operation that assists in the separation of EO components by volatility differences [23]. The large number of compounds in an EO can be separated into a range of isolated fractions. Moreover, vacuum application of fractional distillation allows for a separation avoiding degradation of thermolabile EO compounds, since the system operates at low temperature [24]. In this process, the more volatile compounds are vaporized, followed by intermediate ones, and so on. The less volatile compounds remain in the boiler [24]. Consequently, fractional distillation using a vacuum system would be a helpful tool to separate the compounds of a citrus EO and test their biological properties. Therefore, the aim of this study was to fractionate a commercial citrus EO, Brazilian Orange Terpenes (BOT), using a vacuum distillation system and to characterize the chemical composition, antibacterial, and antioxidant activities of BOT and its separated fractions.

## 2. Results

### 2.1. The Vacuum Fractionation

After the fractional distillation process of BOT oil, four fractions were obtained: F1, F2, F3 and F4. Their yields related to the initial EO carried were 15.2, 34.0, 20.4, and 29.0%, respectively. F1 resulted to be the most volatile fraction, while F4 was the least volatile one (remaining fraction in the boiling flask).

### 2.2. Chemical Composition of the Fractions

**Figure 1:**
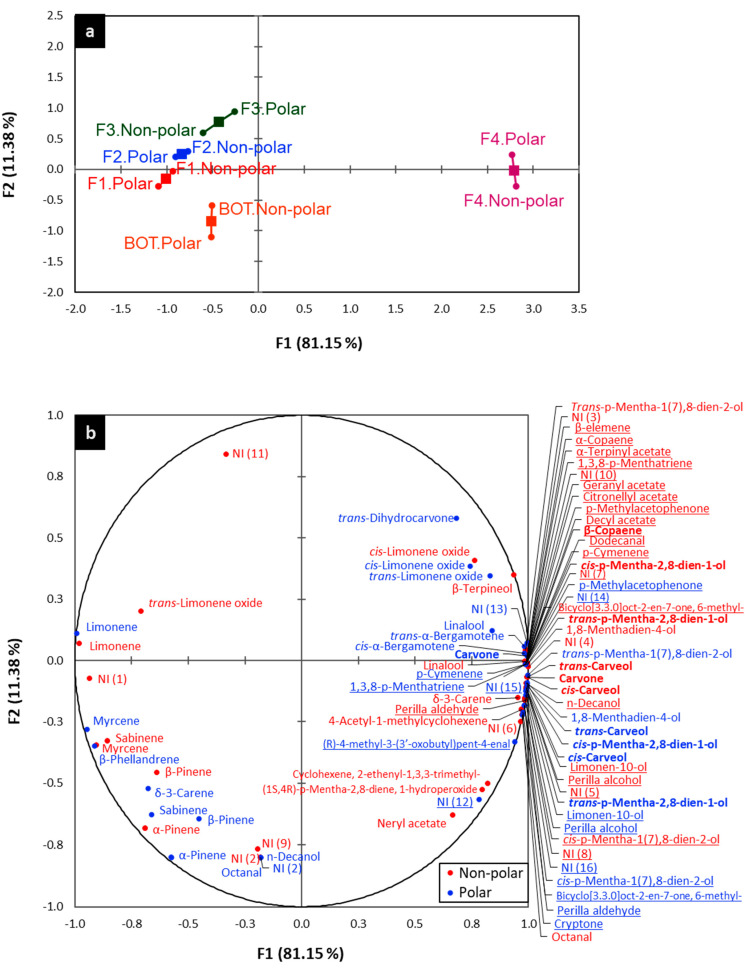
Multiple factor analysis (MFA) of the chemical composition profile of Brazilian orange terpenes’ EO and their fractions. The individual factor map of the overall chemical composition profiles by polar and non-polar identification (**a**) and biplot of the detailed chemical composition profile (**b**).

The full chemical composition of BOT and its fractions (F1, F2, F3, and F4) is shown in Table 1. Overall, the identification of the chemical composition of each fraction and the BOT oil by both columns was quite alike, as is shown in the individual factor map of the MFA (Figure 1a), where the polar and non-polar identification dots were located very close to each other. Furthermore, a very good representation of the chemical composition data was achieved, since the first dimension of the MFA explained 81.15% and the second dimension explained 11.38% of the total variance. As observed in this plot (Figure 1a), the three first fractions—F1, F2 and F3—were practically clustered together, suggesting that these fractions had a very close chemical composition profile. However, they differed from the BOT oil in the second dimension. For these three fractions and the BOT oil, limonene was detected as the major compound; however, fractions were remarkably more associated with this compound in the two dimensions of the MFA than with BOT oil, which had that positive association only with limonene in the first dimension (Figure 1b), thus indicating that F1, F2, and F3 presented higher relative amount of limonene (ranging from 10.86/11.81 to 15.66/16.41) compared to the BOT oil (10.96/13.73) (Table 1).

Nevertheless, F4 presented a fully different chemical profile when compared to the other three fractions (Figure 1a), and it was the fraction that presented a very low relative amount of limonene, 0.13/0.08 (Table 1). F4 was positively associated with a high amount of several compounds, and these are shown in the first and second quadrants of the MFA (Figure 1b). Interestingly, minor compounds such as carvone, *trans*-carveol-, *cis*-carveol, *trans*-p-Mentha-2,8-dien-1-ol, and *cis*-p-Mentha-2,8-dien-1-ol (Figure 1b) were detected in this fraction, and they were present at much higher amount compared to F1, F2 and F3 (Table 1). In addition, others minor compounds were detected exclusively in F4—21 compounds by non-polar GC column and 14 compounds by polar GC column (Figure 1b, underlined names).

### 2.3. Antibacterial Activity and Antioxidant Capacity

Additionally, the biological properties of the BOT oil and its fractions were evaluated. Initially, their antibacterial activity was proved, which is shown in Table 2. Results revealed that F4 was the only fraction that presented an antibacterial effect on the tested bacteria in contrast to the other three fractions (F1, F2, and F3). MIC and MBC values showed that F4 exerted a stronger antibacterial activity on *E. coli* U21 than on the beneficial bacterium *L. rhamnosus*, as observed by the lower MIC/MBC values for *E. coli* U21, which were four times lower than the MIC/MBC for *L. rhamnosus* thus meaning that F4 had a selective antibacterial performance. These results indicated that F4 would be the part of the BOT oil responsible for the observed selective antibacterial activity of the whole BOT oil. However, compared to F4, the BOT oil exhibited higher selective antibacterial activity since it inhibited or killed bacteria at lower concentrations than F4. This suggests the possibility that the resulting antibacterial activity of the BOT oil could be a consequence of the synergism between F4 and the other three fractions.

Subsequently, the BOT oil and its fractions were subjected to screening for their possible antioxidant activity by the ORAC method, as is shown in Table 3. Results showed that F4 exhibited the highest radical scavenging capacity (ORAC value was 844.51 ± 20.20 μmol Trolox/g); however, it was not found to be significantly different from the whole BOT oil. In contrast to the other fractions (F1, F2, and F3), F4 displayed a significant 1.8-, 2.5-, and 3.9-times higher antioxidant activity, respectively. Moreover, compared to the positive controls, α-Tocoferol and BHT, F4 displayed a significant 2.1- and 2.7-times higher antioxidant activity, respectively. Thus, these findings indicate that F4 would be the component of the BOT oil responsible for the resulting antioxidant capacity of the whole BOT oil, as well as for the antibacterial activity already observed.

### 2.4. Relationship between Chemical Composition and Biological Activities

Our results on the biological activity showed that F4 was the part of the BOT oil that presented either the unique or higher antibacterial or antioxidant activities. This would indicate that possibly the minor compounds of BOT oil detected in high amount in F4, such as carvone, *cis*-carveol, *trans*-carveol, *cis*-p-Mentha-2,8-dien-1-ol, and *trans*-p-Mentha-2,8-dien-1-ol would directly influence the selective antibacterial activity and the antioxidant capacity exhibited by BOT oil. Thus, this suggests that the presence of minor compounds could be involved in conferring the biological properties of the BOT oil, instead of this role being attributed exclusively to its major compound, limonene. However, the synergistic interaction between limonene and the minor compounds inside of the BOT oil could also be a determinant for the resulting antibacterial and antioxidant properties of the BOT oil.

## 3. Discussion

Biological properties of citrus EOs have been widely investigated over the last 50 years, focusing on their application in food, cosmetic, and pharmaceutical industries. The recent trend of searching for natural sources of antimicrobials for the food-producing animal sector, such as pig production, has led to considering EOs as a promising alternative to dietary supplementation of antibiotics. However, as a potential alternative to antibiotic growth promoters, it has been highlighted that EOs should comply with a selectivity between pathogenic and beneficial bacteria resident in the animal gut, as a beneficial modulatory effect on pig gut microbiota, in order to improve animal performance [19,20,21]. Interestingly, after an evaluation of 28 EOs obtained from different plants, citrus EOs stood out for presenting a selective antimicrobial activity in the in vitro study by [18]. Complementary and more detailed information about the selectivity of citrus EOS was supplied by [17] when they observed that citrus EOs were shown to cause higher disturbances on the normal growth kinetics of enterotoxigenic *E. coli* isolated from pig gut than on *Lactobacillus* sp. In our study, a citrus EO—BOT—which presented this selective antimicrobial spectrum, was fractionated under vacuum distillation, and our results showed that the BOT oil comprised four fractions with different chemical composition profiles that directly influenced its biological activities.

The fractional distillation process could separate BOT oil into four fractions: F1, F2, F3, and F4 (bottom fraction) using a collection interval of 10 min between fractions. Overall, the relative amount of the compounds present in each obtained fraction varied. Nonetheless, the whole BOT oil was characterized by having limonene as major compound (10.96/13.73 ≈ 78.65/79.38%) [17]. The three first fractions followed this pattern in higher purity (10.86–16.41 ≈ 86.95–93.20%), while the F4 fraction had a very low relative amount of this compound (0.13/0.08 ≈ 0.96/0.61%). These results were in line with a previous study by [23], in which fractional distillation of a green mandarin (*Citrus deliciosa* Tenore) EO allowed the separation of this EO into six fractions removed at the top of the column, and one bottom fraction, using a collection interval of 10 min. The six first fractions as well as the whole mandarin EO were characterized by having limonene as major compound (41.8–75.9%), while the bottom fraction was found to have a lower limonene content (20.1%). Similarly, fractional distillation of a concentrated orange EO left a top fraction rich in limonene and a bottom fraction rich in valencene, with a very low limonene content (0.7–1.9%) [26]. Limonene concentration in our bottom fraction (F4) was quite similar to what was found in this previous study. Moreover, minor compounds of BOT oil such as carvone, *cis*-carveol, *trans*-carveol, *cis*-p-Mentha-2,8-dien-1-ol, and *trans*-p-Mentha-2,8-dien-1-ol were detected at relatively higher amounts in F4 than in F1, F2, and F3 and even than the whole BOT oil. F4 exhibited the greatest biological activities among the fractions and had the lowest limonene content. Therefore, this observation suggests that those compounds would be those mainly responsible for the biological activities exhibited by F4 and consequently by the whole BOT oil. Nonetheless, the stronger biological activities showed by the whole BOT oil also suggest that the synergism between major and minor compounds would determine the biological activities of the citrus EO, BOT, and would be not exclusively determined by its major compound. It has been indicated by [9] that the biological activity of the major compound of an EO is modulated by the minor compounds it contains, forming a synergistic consortium to confer the total biological activities of the EO. For instance, antibacterial activity results showed that fractions rich in limonene (the major compound) did not exhibit any antibacterial effect on the tested bacteria, but F4 in fact presented a selective antibacterial activity, although lower than that of the BOT oil. Because the BOT oil is the combination of the four fractions, it is likely that the higher antibacterial activity of the whole BOT is conferred by the minor compounds in F4, potentiated by their synergistic interaction with limonene inside the BOT oil. This proves that limonene alone would not be exclusively involved in conferring the selective antibacterial activity of this citrus EO. Already, the ineffectiveness of limonene alone on several pathogenic bacteria, including *E. coli*, and beneficial bacteria such as *Lactobacillus* sp. has been reported by [27]. However, some studies have reported some weak antibacterial activity for limonene on *E. coli* and other pathogenic bacteria [28,29]. Nonetheless, on the contrary, a study by Ouwehand et al. (2010) has shown that limonene stimulates the growth of *Lactobacillus* sp. instead of promoting its inhibition or death. Thus, it may be inferred that limonene in the BOT oil would be more related to the lower or non-activity, or even the stimulating activity, of beneficial bacteria, contributing in this way to the selective antibacterial activity of this citrus EO while leaving the function of fighting pathogenic bacteria, such as *E. coli*, to the minor compounds, such as those found in F4. Studies have indicated that the antibacterial effect of citrus EOs on pathogenic bacteria are more related to minor compounds contained in these EOs. These would be mostly oxygenated monoterpenes, which exhibit stronger antibacterial activity than hydrocarbon monoterpenes such as limonene [30,31]. The presence of oxygenated monoterpenes such as carvone, *trans*-carveol, *cis*-p-mentha-2,8-dien-1-ol, *cis*-limonene oxide, and (Z)-patchenol, in higher relative amounts in mandarin EOs than in other Eos, has been suggested as responsible for antibacterial activity of this EO [31]. Indeed, those three first compounds have been detected at relative amounts in F4, and this supports the reasoning that they would be responsible for conferring the antibacterial activity of BOT oil on pathogenic bacteria, such as *E. coli*. Reinforcing this, in previous studies, the wide spectrum activity of carvone alone was observed on several pathogenic bacteria, including several *E. coli* strains [28,32,33]. Moreover, carveol alone has also been a strong antimicrobial compound on gram-positive and gram-negative pathogenic bacteria, such as *E. coli,* inhibiting and killing it at 0.06 and 0.25 mg/mL, respectively [34]. In addition, p-menthadienols compounds in relative amounts, such as *cis*-p-mentha-2,8-dien-1-ol (4.6–9.7%) and *trans*-p-mentha-2,8-dien-1-ol (16.3–22.1%), have been indicated as the responsible compounds for the high antimicrobial activity exhibited by the EOs that contain them [35,36]. The relative content of these two compounds detected in F4 was close to what was found in those previous studies [35,36]. Thus, this confirms the antibacterial role of the five oxygenated monoterpenes detected in BOT as the main responsible compounds for the antibacterial activity observed for F4 and therefore of the citrus EO, BOT, on pathogenic bacteria. Additionally, the other compounds, those exclusively detected in F4, such as geranial [29,30], perrilla aldehyde [37], geranyl acetate [27,29], *cis*-p-mentha-1(7),8-dien-2-ol [35], *cis*-limonene oxide [31], citronellyl acetate, and neryl acetate [27], also would play a contributive role in antibacterial activity. Therefore, the selectivity towards pathogenic bacteria rather than beneficial bacteria by the BOT oil is likely conferred mainly by carvone, *cis*-carveol, *trans*-carveol, *cis*-p-Mentha-2,8-dien-1-ol, and *trans*-p-Mentha-2,8-dien-1-ol in consortia with limonene. The probably mechanism of the selective antibacterial action of BOT oil, induced by the presence of those compounds, would be related to causing higher disturbances of the permeability and integrity of the cytoplasmatic membrane of *E. coli* than *L. rhamnosus*, with the associated higher release of essential cellular constituents [38]. Nevertheless, it is possible that there are other ways whereby those compounds could determine the mode of action of BOT oil on pathogenic bacteria [39].

Similarly, F4 exhibited the greatest free radical scavenging capacity compared to the other three fractions, and this was comparable to the capacity exhibited by the whole BOT oil. It has been observed that increasing concentrations of particular compounds with high antioxidant capacities in a fraction obtained from an EO increases the antioxidant power in that fraction, compared to the antioxidant activity of the EO [40]. This could explain the higher antioxidant capacity of F4 (even though this was not found to be significant). The five major compounds observed in F4 were concentrated, depending on the compound, from 3.2- to 12.1-times higher in comparison to their concentration in the whole BOT oil. These are likely also the responsible compounds for the antioxidant capacity of the BOT oil. This finding agreed with a previous observation by [40], in which the bottom fraction that concentrated some compounds such as carvacrol, trans-caryophyllene, and α-humulene at levels 5.1-, 3.9-, and 5.4-times higher, respectively, exhibited a higher antioxidant capacity than the whole oregano EO. The antioxidant capacity of citrus EOs has been greatly reported and has been attributed mostly to phenolic compounds. However, although monoterpene hydrocarbons and oxygenated monoterpenes comprise the chemical composition of EOs, oxygenated monoterpenes usually more strongly characterize EOs [41,42]. Normally, scarce, if any, antioxidant activity is attributed to monoterpene hydrocarbons [41] such as limonene. For instance, limonene, detected as the most abundant compound present in EOs from Valencia orange and Ponkan and Eureka lemons, was found to display poor antioxidant performance [43]. Thus, this would be the case of the fractions of BOT oil rich in limonene, which exhibited lower antioxidant activity than F4. Conversely, oxygenated monoterpenes were the most present class of compounds in the F4 profile. This chemical class harbors many examples of different functional groups, including phenols, alcohols, aldehydes, ethers, esters, and ketones, with several variants [41]. The antioxidant activity of many of these compounds has already been reported. For instance, alcohol compounds such as carveol and perrilla alcohol, ketones such as carvone, and aldehydes such as perrilla aldehyde have been found to be highly active antioxidant compounds [41] and have been detected either at higher concentrations or exclusively in F4. Moreover, another alcohol compound, geranial (citral), was identified as the highest active antioxidant compound in mandarin EO. Esters such as citronellyl acetate, geranyl acetate, and neryl acetate detected in that EO, individually, showed some antioxidant capacity as well, even more so than limonene [42]. These compounds were also exclusively detected in F4. In addition, ethers such as cis-limonene oxide and trans-limonene oxide have also exhibited moderate antioxidant activity [41]. These two last compounds were also present in F4 at slightly higher amounts than in F1, F2, and F3. Therefore, the presence of all these mentioned compounds in F4 could explain the highest antioxidant performance by F4 and thus would strongly influence the resulting antioxidant activity of the whole BOT oil. A possible synergistic effect between EO compounds on the antioxidant activity could determine the antioxidant mechanism of the whole EO [43]. Hence, dietary supplementation with EOs, such as citrus EOs, could assist the control of biological damages caused by free radical production in the organism, thus offering health benefits against oxidative stress in food-producing animals and also in products derived from these animals by preventing or retarding oxidative activity, for instance, in raw meat [44]. The antioxidant mechanisms of EOs is based on both their ability to donate a hydrogen or an electron to free radicals and their ability to delocalize unpaired electrons within the aromatic structure, thus neutralizing free radicals and protecting other biological molecules against oxidation [22,44]. Due to their antioxidant effect, EOs also affect lipid metabolism in animal tissues by enhancing the antioxidative enzymes activity (superoxide dismutase, catalase, and glutathione peroxidase) and by inhibiting the formation of reactive oxygen species and off-flavors derived from the oxidation of polyunsaturated fatty acids [22]. The beneficial effect produced by the antioxidant capacity of EOs on pigs fed a diet supplemented with EOs has already been observed [45,46,47].

Results of this present study showed that certain compounds could be more involved in conferring the biological activities of the citrus EO, BOT, such as its selective antibacterial activity and its antioxidant potential.

## 4. Materials and Methods

### 4.1. Essential Oils Supply

A commercial citrus EO was used in this study, Brazilian Orange Terpenes (BOT), which was a by-product of orange juice processing and was supplied by a factory in São Paulo State, Brazil. Once the sample was received, it was kept in amber bottles under refrigeration (4 °C) until use. Then, this oil was used as the core material for fractional distillation.

### 4.2. Fractionation of Citrus Essential Oil

The BOT oil was fractionated in a batch vacuum distillation system using a BR–800 Micro fractional distillation apparatus (BR Instruments, Easton, Pennsylvania, USA) equipped with a Teflon spinning band distillation column (20 cm × 7 mm) of 30 theorical plates and covered with a silvered vacuum jacket. The system had an automatic reflux valve (solenoid), coupled at the top of the distillation column, and a condenser (coupled to a condensing bath VWR 1160s, VWR International, Radnor, PA, USA) with a connecting arm linked to a vacuum system and to a fraction collector. This latter instrument allows for the automatic fraction collection according to vapor temperature. The system pressure was regulated by a high-performance vacuum pump (RV3-Edwards, A-VAC Industries, Anaheim, CA, USA) and was monitored by a dual capacitance manometer (PDR 2000, MKS Instruments, Andover, MA, USA). To start the fractional distillation, 20 g of the BOT oil was weighted in a round bottom flask (50 mL) equipped with a thermowell, which allows for to introduction of a temperature probe for controlling the EO temperature during boiling. In addition, the temperature at the top of the fractionating column was also monitored by a thermocouple. The initial temperature in the boiling flask (T_1_) and at the top of the fractionating column (T_2_) were 27 and 23 °C, respectively. Heating of the boiling flasks was performed in a gradual way; thus, T_1_ and T_2_ were constantly monitored. Distillation started once T_1_ reached 54 °C (boiling temperature of BOT), and a first drop of condensed vapor appeared at the end of the condenser. Heat increasing was stopped and maintained at the point where T_1_ and T_2_ were stabilized (approximately 40 min later). Then, the first fraction was collected at: T_1_ = 90 °C; T_2_ = 51 °C; pressure = 17.5 mbar. After 10 min, collection of the first fraction was finished, and immediately following, collection of the second fraction began. Thus, in this experimental protocol, the fractions of BOT oil were collected at regular intervals of 10 min. The remaining fraction in the boiling flask was considered as the bottom fraction, the terpene less one.

### 4.3. Chemical Composition of Essential Oils

The chemical composition characterization of the BOT oil and its fractions was performed by gas chromatography coupled with mass spectrometry (GC/MS) using non-polar and polar columns as described by [17]. BOT oil and its fractions (50 µg) were dissolved in 1mL of dichloromethane containing the internal standard n-tetradecane (0.002% *v*/*v* = 1.38 mg/mL).

The analysis on the non-polar column was carried out in an Agilent Technology gas chromatograph 6890 Plus Series (Santa Clara, CA, USA) coupled to a selective Mass Spectrometry Detector 5973 and an Auto Sampler 7893. A capillary column composed of a fused-silica DB-5MS (J&W Scientific, Folsom, CA, USA) of 60 m × 0.25 mm id × 0.25 μm thick film coated with 5%-phenyl polydimethylsiloxane was used. The oven temperature was set as follows: initial temperature was held at 45 °C for 5 min, then raised to 150 °C at 4 °C/min for 2 min, then raised to 250 °C at 5 °C/min, and finally raised to 30 °C at 10 °C/min, and maintained for 60 min. The injector temperature was 250 °C, with 2 µL of the sample diluted in dichloromethane being injected in “split” mode at a ratio of 30:1. The EIMS electron energy was 70 eV. The mass detector operated in full scan mode with a range from 40 to 350 *m*/*z*. The temperatures of the ion source and transfer line were 230 °C and 285 °C, respectively. Helium gas was used as the carrier gas with an inlet pressure of 16.97 psi. The retention index (RI) was calculated for all the volatile compounds using a homologous series of C7–C30 n-alkanes (49451-U Sigma-Aldrich, Merck KGaA, Darmstadt, Germany), according the linear equation of Van den Dool and Kratz [48].

The analysis on the polar column was carried out in an Agilent Technology gas chromatograph 7890a Plus Series (Palo Alto, CA, USA) coupled to a selective Mass Spectrometry Detector 5975C. A fused-silica capillary column DB-WAX (J&W Scientific, Folsom, CA, USA) of 60 m × 0.25 mm i.d × 0.25 μm thick film coated with polyethylene glycol was used. The oven temperature was set as follows: initial oven temperature was held at 50 °C for 5 min, then raised to 150 °C at 4 °C/min for 7 min, and finally raised to 230 °C at 4 °C/min, and maintained for 40 min. The injector temperature was 250 °C, with 2 µL of the sample diluted in dichloromethane being injected in “split” mode at a ratio of 30:1. The EIMS electron energy was 70 eV. Helium gas was used as the carrier gas with an inlet pressure of 16.91 psi. The mass detector operated in full scan mode ranging from 40 to 350 *m*/*z*.

The retention index (RI) by the polar and non-polar detection was calculated for all the volatile compounds using a homologous series of C7–C30 n-alkanes (49451-U Sigma-Aldrich, St. Louis, MI, USA), according the linear equation of Van den Dool and Kratz [48]. The identification of the components was performed by comparing their RI and mass spectra with data published in the literature [49,50] and in computer libraries (NIST 107 and WILEY 8). The relative peak area of the components was calculated based on internal standardization, that is, using the *n*-tetradecane.

### 4.4. Antibacterial Activity

#### 4.4.1. Bacterial Strains

An ETEC strain and a *Lactobacillus* species were evaluated in this study as models of a pathogenic bacterium and a beneficial bacterium of occurrence in the pig gut, respectively. The ETEC strain, *E. coli* U21 (K88+/LT+/STb+/F18+/Sta+), was isolated from pig gut and provided by The Swine Heath Laboratory of the Department of Preventive Veterinary Medicine and Animal Health, School of Veterinary Medicine and Animal Science at the University of São Paulo, Brazil. *Lactobacillus rhamnosus* ATCC 7469 was purchased from American Type Culture Collection (ATCC). The ETEC strain was cultivated in Tryptic Soy Agar-Difco (TSA) at 37 °C for 18–20 h, and *L. rhamnosus* was grown in MRS (Man, Rogosa and Sharpe) agar at 30 °C for 48 h. After activation, bacteria were sub-cultured in a brain–heart infusion (BHI) or MRS, both supplemented with 15% *v*/*v* of glycerol. After incubation, they were stored at −20 °C until their use.

#### 4.4.2. Determination of Minimal Inhibitory Concentration (MIC)

The determination of the MIC of BOT oil and its fractions was performed by microdilution assay in a 96-well microplate following the standard protocol M07-A9 from the Clinical and Laboratory Standards Institute [51], with some modifications. For the assay, the standard inoculum was prepared in sterile NaCl solution (0.9% *w*/*v*) from living colonies of the selected bacteria, contained in plates of either TSA agar (*E. coli*) or MRS agar (*L. rhamnosus*.) at the optical density equivalent to the 0.5 McFarland Standard (0.08–0.13 at 625 nm). Subsequently, the standard inoculum was diluted at 1:100 to obtain an inoculum of 10^6^ CFU/mL (final inoculum). The stock solution of the BOT and each fraction were prepared at 14.8 mg/mL (1.65% *v*/*v*) with Mueller Hinton (MH) or MRS broth using Tween 80 as emulsifier. From each stock solution, two-fold serial dilutions were made ranging from 14.80 to 0.116 mg/mL. Twenty microliters of the final inoculum were added to each well containing 180 µL of several concentrations of the BOT or its fractions. The final volume in each well was thus 200 µL per well, and the bacterial population was ~10^5^ CFU/mL. The following controls were used: culture medium control (200 µL of MH or MRS broth); growth control (180 µL of MH or MRS broth +20 µL of inoculum) and growth control containing the emulsifier (180 µL of MH or MRS broth with Tween 80 + 20 µL of inoculum). Finally, microplates were incubated at 37 °C for 24 h for *E. coli* and at 30 °C for 36 h for *L. rhamnosus*. The MIC was established as the lowest BOT/fractions concentration that inhibited visible bacterial growth supported by resazurin test at the end of the incubation period. For the resazurin test, 25 µL of resazurin (R7017; Sigma-Aldrich, St. Louis, MI, USA) solution at 0.0135% *m*/*v* was used per well. Then, after visual inspection, the presence of viable cells was evidenced through a change in the resazurin color from blue resazurin to pink resorufin after 1h of further incubation. Assays were carried out in triplicates, with three independent repetitions.

#### 4.4.3. Minimal Bactericidal Concentration (MBC)

The determination of MBC was performed from microplate wells containing concentrations of BOT oil or its fractions where there was no visible bacterial growth. An aliquot of 100 µL was taken from each well and seeded in MH or MRS agar. Then, plates were incubated for 24 h at 37 °C for *E. coli* U21 and for 36 h for *L. rhamnosus*. The MBC was defined as the lowest concentration of BOT/fractions able to cause total bacterial death, represented by the visible absence of colonies on the agar plates.

### 4.5. Antioxidant Capacity

#### 4.5.1. Reagents

Fluorescein (FL), 2,2′-Azobis(2-amidinopropane) dihydrochloride (AAPH), 6-hydroxy-2,5,7,8-tetramethylchroman-2-carboxylic acid (Trolox C), Butil hidroxitolueno (BHT) and α-tocopherol were purchased from Sigma (Sigma-Aldrich, St. Louis, MI, USA). An FL stock solution (8.2 × 10^−4^ mM) was made in a 75 mM potassium phosphate buffer solution (PBS, pH 7.4) and was kept at 4 °C. The AAPH stock solution was prepared in PBS (75 mM, pH 7.4) at a final concentration of 153 mM and was kept in an ice bath. Trolox standard was prepared at 206 μM in PBS (75 mM, pH 7.4), which was the stock solution. From this stock solution, serial dilutions in PBS (75 mM, pH 7.4) were made to reach 10.32, 20.62, 41.23, 61.85, 82.46, and 103.08 μM, which were the working concentrations for the Trolox calibration curve. BHT and α-tocopherol solutions were prepared at 1 mg/mL in PBS as well.

#### 4.5.2. ORAC Assay

The antioxidant capacity of the BOT oil and its fractions was evaluated by the oxygen radical absorbance capacity (ORAC) assessment following the approach used by [52], with some modifications. The assessment was carried out using a Spectrophotometer Modulus™ II Microplate reader, Model 9310-011 (Turner Biosystems Inc., Sunnyvale, CA, USA). For the assessment, FL was used as the fluorescent probe and AAPH as a peroxyl radical generator. BHT and α-tocopherol were used as reference antioxidant compounds.

The BOT oil and its fractions were prepared as follows: 10 mg was accurately weighted, dissolved in 1 mL of methanol and well shaken. After appropriate dilution in PBS (75 mM, pH 7.4), the BOT/fractions working solutions for the analysis were established. Reactions were carried out in a 96-well black microplate, loading 25 µL of the BOT/fractions working solutions and 150 µL of FL solution (8.2 × 10^−4^ mM) into the wells of the microplate. The microplate was pre-incubated at 37 °C for 5 min under stirring into the reader, then 25 µL of AAPH (153 mM) was rapidly added to each well using a multichannel pipet. The microplate was then immediately placed back into the reader and shaken, and the reaction was initiated. Thus, the final reaction mixture was 200 µL and the reduction in fluorescence was recorded by reading fluorescein excitation at 490 nm and emission at 570 nm, every 2.2 min, for 88.6 min. The microplate was automatically shaken prior to each reading. Positive controls—BHT and α-tocopherol—were run in the microplate too. A blank with FL and AAPH using PBS instead of the antioxidant compounds (BHT, α-tocopherol or EO/fractions) was also set in the microplate. Furthermore, the six Trolox calibration concentrations (10.32, 20.62, 41.23, 61.85, 82.46, and 103.08 μM) were placed in the microplate. All reaction mixtures were prepared in octuplicates, and three independent assays were performed for each sample. All fluorescence measurements were expressed relative to the initial reading and plotted vs. time (*x*-axis), generating a typical decay curve. The area under the fluorescence decay curve (AUC) was calculated using the following equation:

(1) $$AUC=1+\overset{i=88.6}{\underset{i=2.2}{\sum }}\frac{f_{i}}{f_{0}}$$

where *f_0_* is the initial fluorescence reading at 0 min and *f_i_* is the fluorescence reading at time *i*. The ORAC value for each sample was calculated by using a linear regression equation between the Net AUC (of Trolox concentrations) and the Trolox concentrations. Thus, ORAC values were expressed as micromole Trolox equivalents (TE) per gram (μmol TE/g). The Net AUC was obtained by subtracting the AUC of the blank from the AUC of Trolox or the AUC of the sample (BOT/fractions).

### 4.6. Data Analysis

The data are shown as the mean of the replicates with the standard deviations. One-way analysis of variance (ANOVA) and Tukey’s test for pairwise comparison at 5% of significance were used to detect significant differences between the antioxidant capacity of the BOT oil and its fractions using R software. In addition, a multiple factor analysis (MFA) was performed on polar and non-polar data (two tables) to describe and contrast the chemical composition profile of the BOT oil and its fractions obtained by GC-MS; this analysis was performed using XLSTAT software.

## 5. Conclusions

Overall, BOT oil fractions differed in terms of their chemical composition profile and their biological activity. The three fractions richest in limonene exhibited lower antioxidant activity compared to the fraction poorest in limonene. The fraction poorest in limonene but richest in other compounds like carvone, *cis*-carveol, *trans*-carveol, *cis*-p-Mentha-2,8-dien-1-ol, and *trans*-p-Mentha-2,8-dien-1-ol showed selective antibacterial activity between pathogenic and beneficial bacteria as well as the highest antioxidant capacity, suggesting that these compounds play an important role in the biological activities observed for this fraction and consequently for the whole BOT oil, instead of to the major compound, limonene, having this function exclusively. Therefore, this highlights the importance of the consortium between minor and major compounds for relevant biological activities of whole EOs. These findings, considered together, represent important information on compounds associated with the bioactivities of a citrus EO, and their future possible application as an antimicrobial/antioxidant agent for food-producing animals, such as pigs.

## Figures and Tables

**Table 1:** Chemical composition of Brazilian orange terpenes’ fractions: F1, F2, F3, and F4.

Compounds ^1^	LRI_c_^2^		LRI_L_^3^		BOT ^6,^*		F1 ^6^		F2 ^6^		F3 ^6^		F4 ^6^	
	NP ^4^	P ^5^	NP	P	NP	P	NP	P	NP	P	NP	P	NP	P
α-Pinene	936	1019	-	1025.4	0.06	0.13	0.06	0.11	0.03	0.05	0.01	-	-	-
Sabinene	975	1118	976	1122	0.04	0.08	0.06	0.10	0.04	0.06	0.02	-	-	-
β-Pinene	981	1105	980	1100	0.01	0.02	0.02	0.03	0.01	-	-	-	-	-
Myrcene	989	1158	991	1160.2	0.12	0.24	0.14	0.28	0.12	0.23	0.06	0.15	-	-
Octanal	1003	-	1002.8	-	0.03	0.002	0.01	-	0.01	-	0.01	-	0.09	-
δ-3-Carene	1015	1146	1011	1146.8	0.02	0.02	0.02	0.03	0.02	0.02	0.01	-	0.07	-
β-Phellandrene	-	1208	-	1209.3	-	0.02	-	0.02	-	0.02	-	0.01	-	-
NI (1)	1028	-	-	-	0.04	-	0.05	-	0.03	-	0.04	-	-	-
Limonene	1037	1201	1031	1198.2	10.96	13.73	11.06	16.41	10.86	15.80	11.81	15.66	0.13	0.08
p-Cymenene	1092	1430	1087.9	1437.5	-	-	-	-	-	-	-	-	0.11	0.08
Linalool	1099	1538	1098	1543.3	0.04	0.06	0.02	0.03	0.03	0.04	0.04	0.07	0.09	0.09
1,3,8-p-Menthatriene	1116	1390	1111	1411	-	-	-	-	-	-	-	-	0.06	0.03
*trans*-p-Mentha-2,8-dien-1-ol	1125	1622	1123	1639	0.12	0.36	0.06	0.09	0.07	0.09	0.13	0.23	1.12	1.31
4-Acetyl-1-methylcyclohexene	1133	-	1137	-	0.02	-	-	-	0.01	-	-	-	0.08	-
*cis*-Limonene oxide	1137	1446	1134	1450.5	0.27	0.33	0.19	0.23	0.22	0.26	0.44	0.50	0.48	0.53
*cis*-p-Mentha-2,8-dien-1-ol	1141	1664	1138	1652.1	-	0.24	-	0.07	-	0.06	-	0.15	1.41	1.15
*trans*-Limonene oxide	1141	1458	1139	1461.6	0.23	0.16	0.13	0.11	0.15	0.12	0.30	0.27	-	0.33
β-Terpineol	1153	-	1157	-	-	-	-	-	0.01	-	0.02	-	0.05	-
Bicyclo [3.3.0]oct-2-en-7-one, 6-methyl-	1172	1694	-	-	-	0.02	-	-	-	-	-	-	0.16	0.10
Cryptone	-	1673	-	1674.8	-	0.02	-	-	-	-	-	-	-	0.08
1,8-Menthadien-4-ol	1183	1680	1189	1681	0.02	0.08	0.01	-	0.01	-	0.02	0.05	0.27	0.40
p-Methylacetophenone	1189	1765	1182.7	1765	-	-	-	-	-	-	-	-	0.07	0.05
*trans*-p-Mentha-1(7),8-dien-2-ol	1191	1789	1180.5	1791	-	0.05	0.01	-	0.01	-	0.02	0.04	0.28	0.28
NI (2)	1192	1732	-	-	0.03	0.48	-	-	-	-	-	-	-	-
NI (3)	1201	-	-	-	0.06	-	0.02	-	0.03	-	0.08	-	0.28	-
NI (4)	1204	-	1205.4	-	0.09	-	0.03	-	0.03	-	0.09	-	0.53	-
*trans*-Carveol	1222	1828	1217	1836.3	0.25	0.40	0.05	0.06	0.04	0.05	0.13	0.20	2.09	2.20
*cis*-p-Mentha-1(7),8-dien-2-ol	1233	1880	1233	1894.9	0.02	0.03	-	-	-	-	-	-	0.12	0.15
*cis*-Carveol	1236	1858	1229	1854.4	0.11	0.18	0.03	0.03	0.02	0.02	0.07	0.09	0.76	0.96
Carvone	1249	1731	1243	1733.6	0.23	0.11	0.04	0.06	0.03	0.04	0.13	0.22	1.77	2.12
n-Decanol	1274	1746	1272.1	-	0.02	0.07	-	-	-	-	-	-	0.21	-
Perilla aldehyde	1281	1781	1273.4	1793.9	0.03	0.04	-	-	-	-	-	-	0.17	0.17
NI (5)	1289	1947	-	-	0.06	-	-	-	-	-	-	-	0.49	-
Limonen-10-ol	1294	1985	1239	1979	0.02	0.03	-	-	-	-	-	-	0.17	0.23
Perilla alcohol	1303	1994	1296.3	2006.6	0.04	0.03	-	-	-	-	-	-	0.35	0.20
Cyclohexene, 2-ethenyl-1,3,3-trimethyl-	1308	-	-	-	0.21	-	0.01	-	-	-	0.03	-	0.30	-
(1S,4R)-p-Mentha-2,8-diene, 1-hydroperoxide	1322	-	-	-	0.20	-	-	-	-	-	0.02	-	0.27	-
NI (6)	1335	-	-	-	0.13	-	-	-	-	-	0.02	-	0.40	-
NI (7)	1344	-	-	-	-	-	-	-	-	-	-	-	0.12	-
Citronellyl acetate	1349	-	1352.4	-	-	-	-	-	-	-	-	-	0.25	-
α-Terpinyl acetate	1357	-	1347	-	-	-	-	-	-	-	-	-	0.14	-
Neryl acetate	1360	-	1362.9	-	0.21	-	-	-	-	-	0.02	-	0.21	-
NI (8)	1367	-	-	-	0.04	-	-	-	-	-	-	-	0.24	-
Geranyl acetate	1375	-	1381	-	-	-	-	-	-	-	-	-	0.24	-
NI (9)	1377	-	-	-	0.16	-	-	-	-	-	0.01	-	-	-
α-Copaene	1386	-	1377	-	-	-	-	-	-	-	-	-	0.13	-
NI (10)	1393	-	-	-	-	-	-	-	-	-	-	-	0.16	-
β-elemene	1396	-	1390.4	-	-	-	-	-	-	-	-	-	0.13	-
Dodecanal	1409	-	1408.1	-	-	-	-	-	-	-	-	-	0.19	-
Decyl acetate	1416	-	1407.1	-	-	-	-	-	-	-	-	-	0.11	-
*cis*-α-Bergamotene	1420	1552	1414.5	1559.1	-	0.11	-	0.03	-	0.05	-	0.13	-	0.34
*trans*-α-Bergamotene	1440	1559	1434.5	1575.7	-	0.06	-	0.06	-	0.03	-	0.09	-	0.26
β-Copaene	1447	-	1433.1	-	-	-	-	-	-	-	-	-	1.10	-
NI (11)	1701	-	-	-	-	-	-	-	0.02	-	0.03	-	-	-
*trans-*Dihydrocarvone	-	1609	-	1623.1	-	-	-	-	-	-	-	0.03	-	0.03
NI (12)	-	1633	-	-	-	0.03	-	-	-	-	-	-	-	0.04
NI (13)	-	1746	-	-	-	-	-	-	-	-	-	0.05	-	0.43
NI (14)	-	1839	-	-	-	-	-	-	-	-	-	-	-	0.34
NI (15)	-	1919	-	-	-	-	-	-	-	-	-	-	-	0.10
(R)-4-methyl-3-(3′-oxobutyl)pent-4-enal	-	1946	-	-	-	0.10	-	-	-	-	-	-	-	0.26
NI (16)	-	1978	-	-	-	0.01	-	-	-	-	-	-	-	0.05

^1^ Identification by GC/MS using non-polar and polar columns. ^2^ LRI_C_: Linear retention index (Calculated). ^3^ LRI_L_: Linear retention index (Literature). ^4^ NP: non-polar column DB-5MS. ^5^ P: polar column DB-WAX. ^6^ Relative amounts of the identified compounds on internal standardization using n-tetradecane as internal standard (0.002 *v*/*v* or 1.38 mg/mL). *Data published previously in [25]. NI: Not identified compound.

**Table 2:** Antibacterial activity of BOT oil and its fractions.

EO/Fractions	Antibacterial Parameter (mg/mL)	Bacterial Strain	
		*E. coli* U21	*L. rhamnosus* ATCC 7469
F1	MIC	-	-
	MBC	-	-
F2	MIC	-	-
	MBC	-	-
F3	MIC	-	-
	MBC	-	-
F4	MIC	3.70	14.80
	MBC	3.70	14.80
BOT *	MIC	1.85	3.70
	MBC	1.85	7.40

* Values determined in the study by [17].

**Table 3:** Antioxidant capacity of the BOT oil and its fractions according to the ORAC assay.

EO/Fractions	Antioxidant Capacity µmol Trolox/g of Pure Sample
F1	477.64 ± 35.45 ^b^
F2	343.24 ± 26.57 ^c^
F3	211.69 ± 14.73 ^d^
F4	850.67 ± 16.04 ^a^
BOT	785.14 ± 79.35 ^a^
α-Tocoferol	339.21 ± 14.55 ^c^
BHT	312.16 ± 11.02 ^c^

* Values are means ± standard deviation (SD) of triplicate determinations. a–d: Mean values within a column having different superscripts are significantly by the least significant difference Tukey test (*p* < 0.05).

## Data Availability

Data present in this study are available on request from the corresponding author.

## Sample Availability

Samples of the essential oils are available from the authors on request.
